# Proteomics analysis of urine reveals acute phase response proteins as candidate diagnostic biomarkers for prostate cancer

**DOI:** 10.1186/s12953-014-0059-9

**Published:** 2015-01-29

**Authors:** Katarina Davalieva, Sanja Kiprijanovska, Selim Komina, Gordana Petrusevska, Natasha Chokrevska Zografska, Momir Polenakovic

**Affiliations:** Research Centre for Genetic Engineering and Biotechnology “Georgi D Efremov”, Macedonian Academy of Sciences and Arts, Krste Misirkov 2, 1000 Skopje, Republic of Macedonia; Institute of Pathology, Medical Faculty, University “St. Cyril and Methodius”, Skopje, Republic of Macedonia; Biochemical laboratory, Clinical Hospital “Acibadem Sistina”, Skopje, Republic of Macedonia

**Keywords:** Prostate cancer, Benign prostate hyperplasia, 2-D DIGE, MS, Urine analysis, Non-invasive biomarkers

## Abstract

Despite the overall success of prostate specific antigen (PSA) in screening and detection of prostate cancer (PCa), its use has been limited due to the lack of specificity. The principal driving goal currently within PCa research is to identify non-invasive biomarker(s) for early detection of aggressive tumors with greater sensitivity and specificity than PSA. In this study, we focused on identification of non-invasive biomarkers in urine with higher specificity than PSA. We tested urine samples from PCa and benign prostatic hyperplasia (BPH) patients by 2-D DIGE coupled with MS and bioinformatics analysis. Statistically significant (p < 0.05), 1.8 fold variation or more in abundance, showed 41 spots, corresponding to 23 proteins. The Ingenuity Pathway Analysis showed significant association with the Acute Phase Response Signaling pathway. Nine proteins with differential abundances were included in this pathway: AMBP, APOA1, FGA, FGG, HP, ITIH4, SERPINA1, TF and TTR. The expression pattern of 4 acute phase response proteins differed from the defined expression in the canonical pathway. The urine levels of TF, AMPB and HP were measured by immunoturbidimetry in an independent validation set. The concentration of AMPB in urine was significantly higher in PCa while levels of TF and HP were opposite (p < 0.05). The AUC for the individual proteins ranged from 0.723 to 0.754. The combination of HP and AMBP yielded the highest accuracy (AUC = 0.848), greater than PSA. The proposed biomarker set is quickly quantifiable and economical with potential to improve the sensitivity and specificity of PCa detection.

**Electronic supplementary material:**

The online version of this article (doi:10.1186/s12953-014-0059-9) contains supplementary material, which is available to authorized users.

## Background

The introduction of prostate specific antigen (PSA) as a biomarker for prostate cancer (PCa) screening and detection has transformed the management of this disease [1-3]. Despite the overall success of the PSA blood test, its use has been limited due to the lack of specificity, especially in patients with total serum PSA levels in a range of 2–10 ng/ml. Various nonmalignant processes such as benign prostatic hyperplasia (BPH) and prostatitis, as well as manipulation and medical interventions of the prostate lead to serum PSA elevations and subsequently limit the specificity of PSA for cancer detection [4]. Additionally, 15% of PCa cases occur in men with normal serum PSA levels [5]. These data have encouraged considerable investigation into the search for novel PCa biomarkers.

The rise of –omics technologies in recent years and their use in PCa research has delivered a number of new potential biomarkers for PCa [6-8]. These included proteins, fusing genes, RNA transcripts and epigenetic modifications of DNA. Among the available technologies, proteomics has shown large potential in identification of PCa biomarkers [9,10]. As a result of the found differences in protein expression profiles between BPH and PCa, and among different types and grades of cancer, a number of proteins in tissue and biological fluids (serum, plasma, urine, seminal plasma) were identified as potential diagnostic or prognostic markers for PCa. Prostate tissue, although a rich source of potential PCa biomarkers, has the most invasive sampling, low tolerability and carries significant morbid risk [11]. On the other hand, a screening procedure based on biological fluid testing is highly desirable because of the minimally invasive and low-cost procedures for collecting samples. Hence, the current extensive investigation in PCa biomarkers is mostly oriented to the identification of highly specific non-invasive and easily accessible biomarkers in urine, seminal plasma and minimally invasive blood samples.

The number of newly identified diagnostic PCa biomarker candidates in biological fluids is rising over the time. Those found in serum include various precursor forms of PSA [12,13], α-2-macroglobulin [14,15], Zinc-α2-glycoprotein [14,16,17], Pigment epithelium-derived factor [16,18], Eukaryotic translation elongation factor 1 alpha 1, Fibronectin 1 [14], Chemokine ligand 16, Pentraxin 3, Spondin 2, Follistatine [19,20], panels of serum proteins [21] and many other soluble factors and intracellular proteins involved in structural or metabolic functions. Candidates for urine biomarkers include Annexin A3 [22], Inter-alpha-trypsin inhibitor heavy chain 4 [23], CD90 [24], Calgranulin/MRP-14 [25], Semenogelin 1, Uromodulin [26] and Engrailed-2 [27]. Some of these proteins have been identified in independent studies with different proteomics methods and their usefulness is yet to be validated in a large cohort within and across different ethnic populations. However, most of the data obtained until now is quite heterogeneous and there is a small percentage of overlap between independent studies. No single test or proposed biomarker to date can fulfill the requirements for the ideal PCa diagnostic biomarker and the next PCa screening tool. Furthermore, it is becoming clearer that the ideal PCa diagnostics test will most likely be based not on a single but multiple biomarkers, due to the clinical heterogeneity of the cancer and the need to distinguish the disease from greatly prevalent inflammatory and benign conditions [6,8]. This highlights the necessity of future extensive comparative analysis of well-defined samples for identification of a reliable diagnostic PCa biomarker or biomarker panel.

The aims of this study were to characterize the pattern of differential protein abundances in urine of PCa and BPH patients using two-dimensional difference in gel polyacrylamide gel electrophoresis (2-D DIGE) coupled with mass spectrometry (MS) and to identify a biomarker or marker panel for non-invasive PCa diagnosis preferentially with greater specificity and sensitivity from the ones that are currently in use. Another objective was to compare our results with those of other published studies and to assess the level of compatibility across different technological platforms used and different ethnic background of samples. The identified proteins with differential abundances between the PCa and BPH groups from this study were significantly associated with the Acute Phase Response Signaling pathway. We have been able to successfully validate these findings by confirming the differential abundance of TF, HP and AMBP in urine in an independent validation set. The results from this study may provide a clinically useful diagnostic set for the screening and detection of PCa.

## Results

### 2-D DIGE analysis

**Figure 1:**
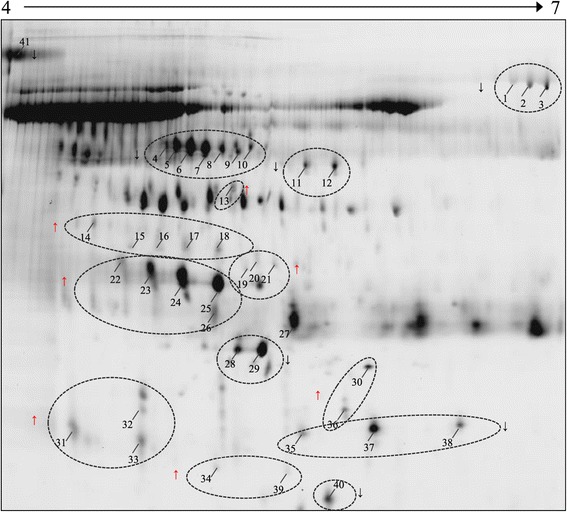
**Representative 2-D map of the urine proteome obtained by using IEF on pH 4–7 IPG strip and 2-D gel electrophoresis on 12.5% SDS-PAGE.** All proteins with differential abundance between studied groups are marked with numbered arrows. Details of these proteins identified by MALDI MS are tabulated in Table 1. Proteins with increased abundance in PCa are marked with red arrows.

The number of the detected spots in the DeCyder DIA workspaces in all gels ranged from 1308 to 1577. In the BVA module, the spots were matched among 4 gels with an average of 1063 matched spots. One hundred and thirty four spots showed statistically significant (p < 0.05) 1.8 fold variation or more in abundance. However, upon manual checking of the spot quality, the majority of the protein spots with differential abundance were either part of the most abundant protein in the urine, later identified as albumin [28], or were low quality spots. Therefore, only 41 spots were selected for further analysis (Figure 1). All of these spots fulfilled the criteria for presence in all spot maps. Among these, 22 spots showed higher abundance in the PCa group (up-regulated) and 19 spots had lower abundance in PCa (down-regulated).

**Figure 2:**
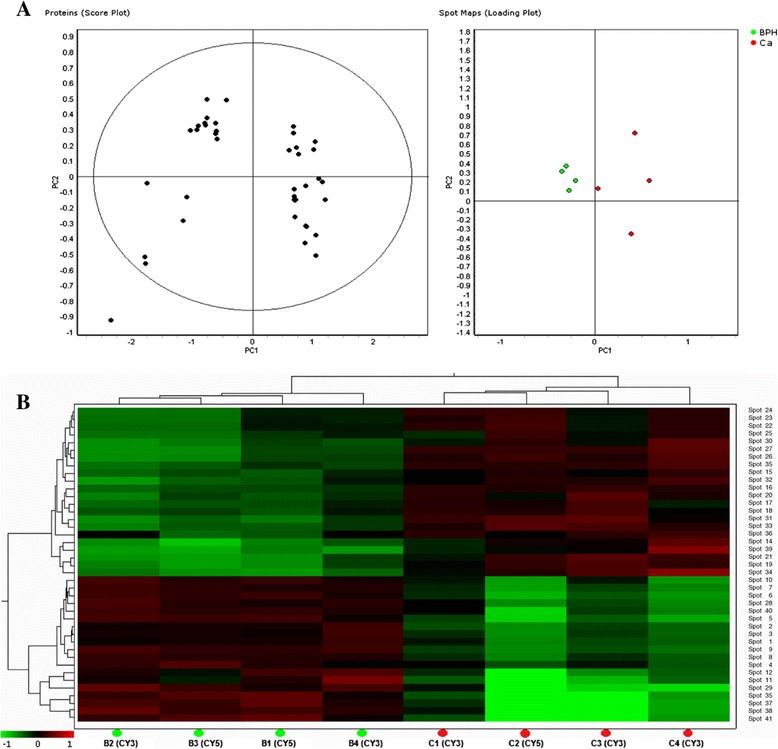
**Principal component analysis and hierarchical cluster analysis of the proteins with differential abundance. (A)** Scatter plots of the principal component analysis where green dots represent urine samples from BPH patients and red dots samples from PCa patients. **(B)** The hierarchical clustering result: higher abundance in PCa group is coloured in red, the lower ones in green. Column descriptors indicate the 4 samples per group (B = BPH; C = PCa) and the labeling dye, while the row descriptors indicate proteins with their spot numbers (given in Table 1). The dendrograms represent the distances between the clusters.

Principle component analysis and hierarchical clustering analysis of the 41 spots with differential abundance were performed by EDA module, available within the DeCyder software (Figure 2). Two-dimensional scatter plots of the principal components of urine samples showed a good clear separation between samples from PCa and BPH patients (Figure 2A). Hierarchical clustering analysis (Figure 2B) showed that, based on the abundance pattern of the 41 spots, samples from PCa and BPH groups form two distinct separate clusters.

### Identification and interpretation of proteins with differential abundance

All of the 41 spots with differential abundance were identified by MALDI-MS. Fold changes of the 41 identified protein spots in the two groups along with the detailed Mascot search results are given in Table 1. Several proteins were identified in multiple spots, most likely due to posttranslational modification leading to shifts in the 2-D. So, the identified spots with differential abundance equaled to 23 distinct proteins, among which 14 were up-regulated and 9 were down-regulated in PCa.

**Table 1:** **List of proteins with differential abundance identified by MALDI-MS**

**Spot No.**	***T*** **-test**	**Fold Ratio (PCa/ BPH)**	**Protein name**	**Swiss-Prot accession No.**	**Mw (kDa)**	**pI**	**Mascot protein score**	**RMS error (ppm)**	**p value**	**No. of matched peptides out of total**	**% of sequence coverage**
1	0.010	−2.53	Serotransferrin	TRFE_HUMAN	79.29	6.81	98	80	3.1E-06	10/15	21
2	0.010	−2.64	Serotransferrin	TRFE_HUMAN	79.29	6.81	200	47	2.0E-16	23/43	38
3	0.010	−2.64	Serotransferrin	TRFE_HUMAN	79.29	6.81	243	57	1.0E-20	22/30	40
4	0.033	−2.17	Alpha-1-antitrypsin	A1AT_HUMAN	46.89	5.37	81	56	1.9E-04	12/31	31
5	0.002	−5.21	Alpha-1-antitrypsin	A1AT_HUMAN	46.89	5.37	128	82	3.2E-09	18/34	36
6	0.002	−4.34	Alpha-1-antitrypsin	A1AT_HUMAN	46.89	5.37	194	51	8.1E-16	23/43	53
7	0.002	−3.66	Alpha-1-antitrypsin	A1AT_HUMAN	46.89	5.37	74	109	7.7E-04	12/28	27
8	0.001	−3.05	Alpha-1-antitrypsin	A1AT_HUMAN	46.89	5.37	178	63	3.2E-14	20/31	41
9	0.000	−4.27	Alpha-1-antitrypsin	A1AT_HUMAN	46.89	5.37	66	102	4.9E-03	9/17	26
10	0.010	−3.18	Vitamin D-binding protein	VTDB_HUMAN	54.52	5.40	135	65	6.4E-10	12/24	50
11	0.011	−6.11	Fibrinogen gamma chain	FIBG_HUMAN	52.10	5.37	164	108	8.1E-13	17/30	47
12	0.019	−4.41	Thymidine phosphorylase	TYPH_HUMAN	50.32	5.36	64	104	7.4E-03	5/13	25
13	0.036	2.59	Gelsolin	GELS_HUMAN	86.04 (52.48)	5.90 (5.34)	80	81	2.0E-03	9/15	16
14	0.004	4.75	Fibrinogen alpha chain (fragment)	FIBA_HUMAN	95.65 (~50)	5.70 (4.65)	91	99	1.6E-05	10/18	15
15	0.003	2.47	Endonuclease domain-containing 1 protein	ENDD1_HUMAN	55.72	5.55	104	122	8.1E-07	9/13	20
16	0.000	3.07	E3 ubiquitin-protein ligase rififylin	RFFL_HUMAN	41.74	5.33	59	97	2.7E-02	5/12	26
17	0.009	2.72	Inter-alpha-trypsin inhibitor heavy chain H4 (fragment)	ITIH4_HUMAN	103,52 (~45)	6,51 (5,00)	64	51	9.1E-03	13/26	12
18	0.004	2.66	Inter-alpha-trypsin inhibitor heavy chain H4 (fragment)	ITIH4_HUMAN	103.52 (~45)	6.51 (5.15)	88	134	2.9E-05	11/26	13
19	0.001	4.75	Quinone oxidoreductase-like protein 1	QORL1_HUMAN	39.07	5.49	66	91	4.9E-02	4/12	12
20	0.005	3.17	Interleukin enhancer-binding factor 2	ILF2_HUMAN	43.26	5.19	62	82	3.0E-02	5/13	19
21	0.002	5.07	Vesicular integral-membrane protein VIP36	LMAN2_HUMAN	40.54	6.46	80	75	1.9E-04	7/14	21
22	0.041	2.26	Protein AMBP	AMBP_HUMAN	39.87	5.95	86	15	5.5E-05	8/15	22
23	0.048	2.25	Protein AMBP	AMBP_HUMAN	39.87	5.95	93	78	9.9E-06	10/24	23
24	0.044	2.26	Protein AMBP	AMBP_HUMAN	39.87	5.95	94	109	8.4E-06	10/19	24
25	0.035	2.35	Protein AMBP	AMBP_HUMAN	39.87	5.95	130	93	2.0E-09	15/35	37
26	0.023	3.33	Prostaglandin-H2 D-isomerase	PTGDS_HUMAN	21.24	7.66	61	52	1.8E-02	7/17	32
27	0.033	3.43	Prostaglandin-H2 D-isomerase	PTGDS_HUMAN	21.24	7.66	64	23	7.7E-03	8/36	33
28	0.005	−3.25	Apolipoprotein A-I	APOA1_HUMAN	30.75	5.56	116	57	5.1E-08	16/53	46
29	0.010	−6.82	Apolipoprotein A-I	APOA1_HUMAN	30.75	5.56	171	74	1.6E-13	20/55	57
30	0.009	3.55	Basement membrane specific heparan sulphate proteoglycan core protein (PERLECAN) (fragment)	PGBM_HUMAN	468.83 (20.65)	6.06 (5.47)	204	62	1.1E-15	15/33	88
31	0.001	4.60	CD59 glycoprotein	CD59_HUMAN	14.79	6.02	58	103	2.9E-02	4/7	21
32	0.006	3.11	CD59 glycoprotein	CD59_HUMAN	14.79	6.02	58	32	2.9E-02	4/7	21
33	0.000	4.17	CD59 glycoprotein	CD59_HUMAN	14.79	6.02	61	47	1.6E-02	5/15	28
34	0.001	6.56	Secreted and transmembrane protein 1	SCTM1_HUMAN	27.30	7.00	66	34	4.9E-03	6/13	22
35	0.019	−5.51	Haptoglobin (fragment, alpha 2 chain)	HPT_HUMAN	45.86 (~16)	6.13 (6.20)	73	79	9.3E-03	4/11	28
36	0.017	2.26	Mannan-binding lectin serine protease 2 (chainA, Human MBL associated protein 19)	MASP2_HUMAN	77.19 (19.53)	5.39 (5.44)	127	24	5.5E-08	7/14 (8/14)	37
37	0.015	−5.86	Haptoglobin (fragment, alpha 2 chain)	HPT_HUMAN	45.86 (~16)	6.13 (6.20)	88	32	7.5E-04	7/18	34
38	0.024	−6.58	Haptoglobin (fragment, alpha 2 chain)	HPT_HUMAN	45.86 (~16)	6.13 (6.20)	80	41	1.9E-04	11/34	31
39	0.004	6.21	Secreted and transmembrane protein 1	SCTM1_HUMAN	27.30	7.00	75	47	6.1E-04	6/9	22
40	0.012	−3.39	Transthyretin	TTHY_HUMAN	15.99	5.52	160	32	2.0E-12	10/27	73
41	0.010	−3.54	Uromodulin	UROM_HUMAN	72.64	5.05	158	57	3.2E-12	18/24	22

The 23 identified proteins were grouped into different classes based on sub cellular and functional information available (Additional file 1: Figure S1). The majority of the proteins were enzymes (30%), transporters (22%), enzyme inhibitors (13%) and proteins of unknown function (17%). Most of the proteins were secreted (74%), 17% were cytoplasmic and only 8% membrane or nucleus proteins. Binding was the major molecular function (50%), followed by catalytic activity (27%) and transport (15%). The GO based data of biological processes pointed to a variety of processes in which the identified proteins are putatively involved. Detailed information regarding biological function is given in Table 2.

**Table 2:** **Functional characterization of the proteins with differential abundance between PCa and BPH and association with urogenital cancers**

**Protein name**	**Symbol**	**Expression (PCa/BPH)**	**Biological function**	**Association with PCa** ^**a**^	**Association with urogenital cancers** ^**a**^
Serotransferrin	TF	Down-regulated	Iron transport, acute-phase response, stimulation of cell proliferation	Down-regulated, serum [21]; Down-regulated, urine [29]	
Alpha-1-antitrypsin	SERPINA1	Down-regulated	Blood coagulation, acute phase response, hemostasis	Differentially expressed, tissue [30]; Up-regulated, serum [31]; Down-regulated, serum [21]	Up-regulated, urine, bladder cancer [32];
Vitamin D-binding protein	GC	Down-regulated	Vitamin D metabolic process, vitamin transport, transmembrane transport	Differentially expressed, tissue [33]	
Fibrinogen gamma chain	FGG	Down-regulated	Cell activation, protein complex assembly, response to stress, signal transduction, blood coagulation, hemostasis	Differentially expressed, tissue [30]	Degradation products in urine - markers for bladder cancer [34]; Up-regulated, urine, bladder cancer [35]
Thymidine phosphorylase	TYMP	Down-regulated	DNA replication, angiogenesis, response to stimulus	Potential marker for PCa [28]	Up-regulated, tissue, renal cell carcinoma [36]
Gelsolin	GSN	Up-regulated	Promote the assembly of monomers into filaments, transport, apoptosis, response to stress	Differentially expressed, tissue [33]; Down-regulated, tissue [37]	
Fibrinogen alpha chain	FGA	Up-regulated	Cell activation, protein complex assembly, response to stress, signal transduction, blood coagulation, hemostais		Degradation products in urine - markers for bladder cancer [34]; Up-regulated, urine, bladder cancer [35]
Endonuclease domain-containing 1 protein	ENDOD1	Up-regulated	Unknown, may act as DNase/RNase	Potential marker for PCa [28]	
E3 ubiquitin-protein ligase rififylin	RFFL	Up-regulated	Protein transport, proteolysis, apoptosis	Potential marker for PCa [28]	
Inter-alpha-trypsin inhibitor heavy chain H4	ITIH4	Up-regulated	Acute inflammatory response, carbohydrate metabolic process, nitrogen compound metabolic process	Up-regulated, urine [23];	
				Down-regulated, serum [31]	
Quinone oxidoreductase-like protein 1	CRYZL1	Up-regulated	Quinone cofactor metabolic process, cellular metabolic process	Potential marker for PCa [28]	
Interleukin enhancer-binding factor 2	ILF2	Up-regulated	Immune response, regulation of transcription, nucleobase-containing compound metabolic process	Potential marker for PCa [28]	
Vesicular integral-membrane protein VIP36	LMAN2	Up-regulated	Protein localization, protein transport, macromolecule localization		
Protein AMBP (Alpha-1-microglobulin/ bikunin precursor)	AMBP	Up-regulated	Cell adhesion, immune response, regulator of biological processes, regulation of signal transduction	Up-regulated, urine [23];	
				Down-regulated, urine [38]; Down-regulated, serum [21]	
Prostaglandin-H2 D-isomerase	PTGDS	Up-regulated	Fatty acit metabolism, lipid biosynthesis, transport	Up-regulated, urine [38]	
Apolipoprotein A-I	APOA1	Down-regulated	Regulation of cytokine production, regulation of lipid transport, lipid metabolism	Differentially expressed, tissue [30]; Down-regulated, serum [31]; Down-regulated, serum [21]	Up-regulated, urine, bladder cancer [35]
Laminin G Like Domain 3 From Human Perlecan	HSPG2	Up-regulated	Developmental processes, cell adhesion, cell differentiation, localization		
CD59 glycoprotein	CD59	Up-regulated	Response to stress, cell surface receptor linked signal transduction, blood coagulation, hemostasis, response to external stimulus		
Secreted and transmembrane protein 1	SECTM1	Up-regulated	Immune response, regulation of signal transduction, multicellular organismal development		
Haptoglobin (α-chain)	HP	Down-regulated	Proteolysis, cellular iron ion homeostasis, response to stress, defense response, metabolic process	Down-regulated, urine [38];	Up-regulated, urine, bladder cancer [35]
				Down-regulated, tissue [39]; Down-regulated, serum [21]	
Mannan-binding lectin serine protease 2	MASP2	Up-regulated	Complement activation, lectin pathway, activation of immune response		
Transthyretin	TTR	Down-regulated	Transport, localization, establishment of localization	Up-regulated, serum [31]	
Uromodulin	UMOD	Down-regulated	Response to stress, cellular defense response, regulation of cell proliferation	Down-regulated, urine [26]	

^a^Based on published proteomics studies retrieved from PubMed.

**Figure 3:**
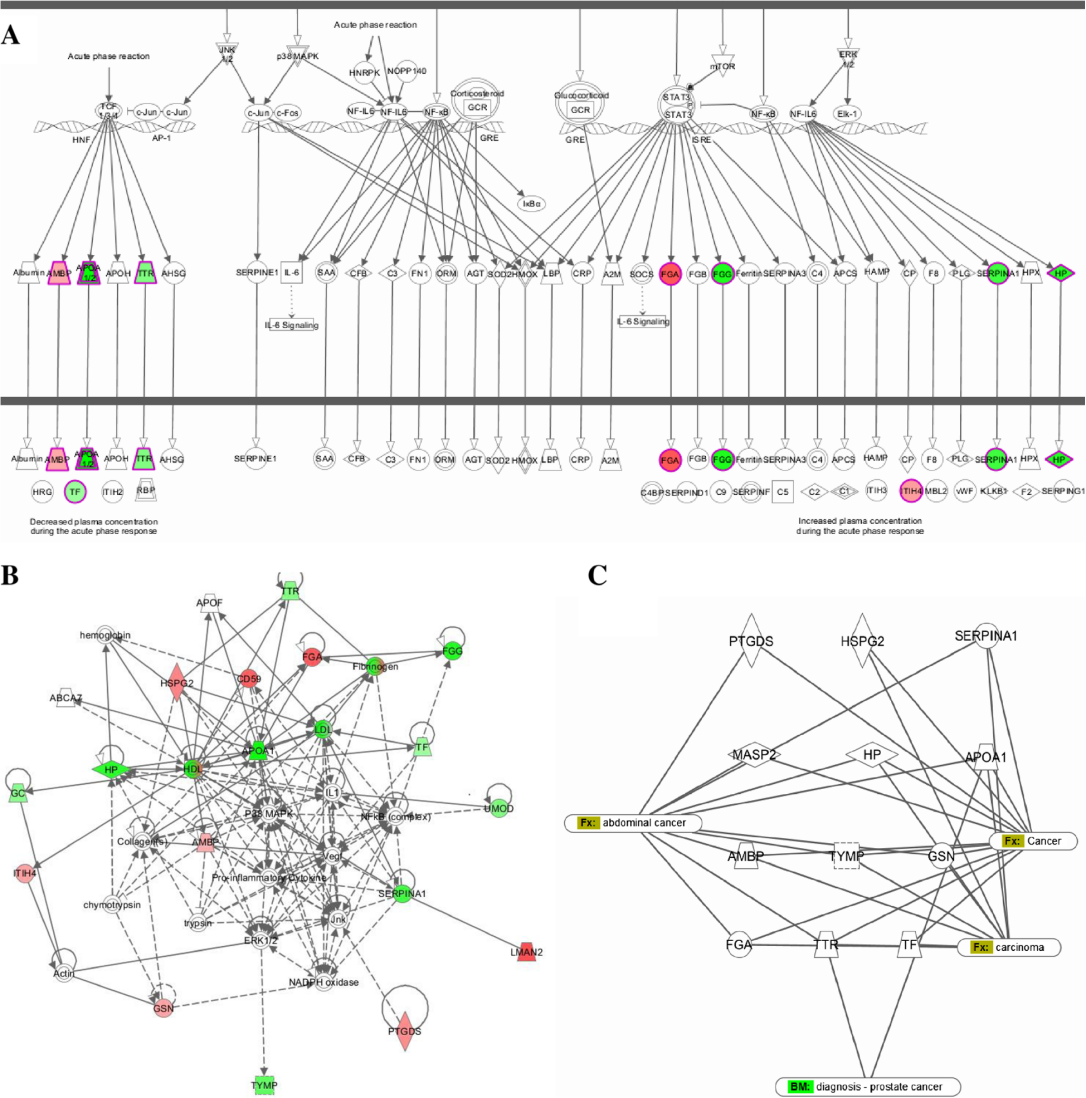
**Pathways and networks associated with proteins with differential abundance according to IPA. (A)** The top canonical pathway significantly associated with the differentially expressed proteins - Acute Phase Response Signaling (p = 6,99 e^−14^). **(B)** Highest ranked protein network of functional associations between 23 proteins with differential abundance - Cancer, Organism injury and abnormalities and Gastrointestinal Disease. Most of the proteins with differential abundance are closely connected in the network through three major nodes: P38 MARK, Pro-inflamatory cytokine and ERK1/2. The network is graphically displayed with proteins as nodes and the biological relationships between the nodes as lines. The color of the shapes indicates the degree of over-expression (red) or under-expression (green) of the corresponding protein in PCa compared to BPH samples. Direct connection between molecules is represented by a solid line and indirect connection by broken line. The length of a line reflects published evidence supporting the node-to-node relationship concerned. **(C)** Selected subset of proteins with differential abundance associated with cancer in humans or cancer cell lines.

The IPA analysis of associations between our set of proteins and known biological pathways showed significant association with the Acute Phase Response Signaling pathway (p = 6,99E-14). Nine of the 23 proteins with differential abundance are included in this pathway: α-1-microglobulin/bikunin (AMBP), apolipoprotein A-I (APOA1), fibrinogen alpha chain (FGA), fibrinogen gamma chain (FGG), haptoglobin (HP), inter-alpha-trypsin inhibitor (ITIH4), alpha-1-antitrypsin (SERPINA1), transferrin (TF) and transthyretin (TTR) (Figure 3A).

According to the IPA functional classification - Diseases and Disorders, 9 of our proteins (APOA1, CD59, FGG, GSN, HSPG2, MASP2, PTGDS, SERPINA1 and TYMP) are significantly associated with the category - Organism Injury and Abnormalities, sub category – Lesion formation (p = 9,05E-10) while in Molecular and Cellular Function category, 14 proteins (APOA1, CD59, FGA, GSN, HP, HSPG2, ILF2, ITIH4, PTGDS, SERPINA1, TF, TTR, UMOD and TYMP) were found significantly associated with cell death (p = 2,29E-5).

The highest ranked protein network of functional associations between the differentially expressed proteins according to IPA was Cancer, Organism Injury and Abnormalities and Gastrointestinal Disease (score 46) (Figure 3B). Seventeen out of 23 proteins with differential abundance (TTR, HSPG2, CD59, FGA, FGG, GC, HP, APOA1, TF, ITIH4, AMBP, UMOD, SERPINA1, GSN, TYMP, PTGDS and LMAN2) are closely connected in the network through three major nodes: P38 Mitogen-activated protein kinase (P38 MARK), extracellular-signal-regulated kinase 1/2 (ERK1/2) and pro-inflamatory cytokines.

Our set of proteins was further analyzed using IPA biomarker filter which allows matching the input protein list with known disease profiles consisting of maps, networks and lists of biomarkers known for a disease. Results revealed that 12 of the proteins (AMBP, APOA1, FGA, GSN, HP, HSPG2, MASP2, PTGDS, SERPINA1, TF, TTR and TYMP) are predicted markers for prostate cancer (Figure 3C). The majority of these proteins (7/12) are also part of the Acute Phase Response Signaling pathway.

### Validation of candidate biomarkers

**Figure 4:**
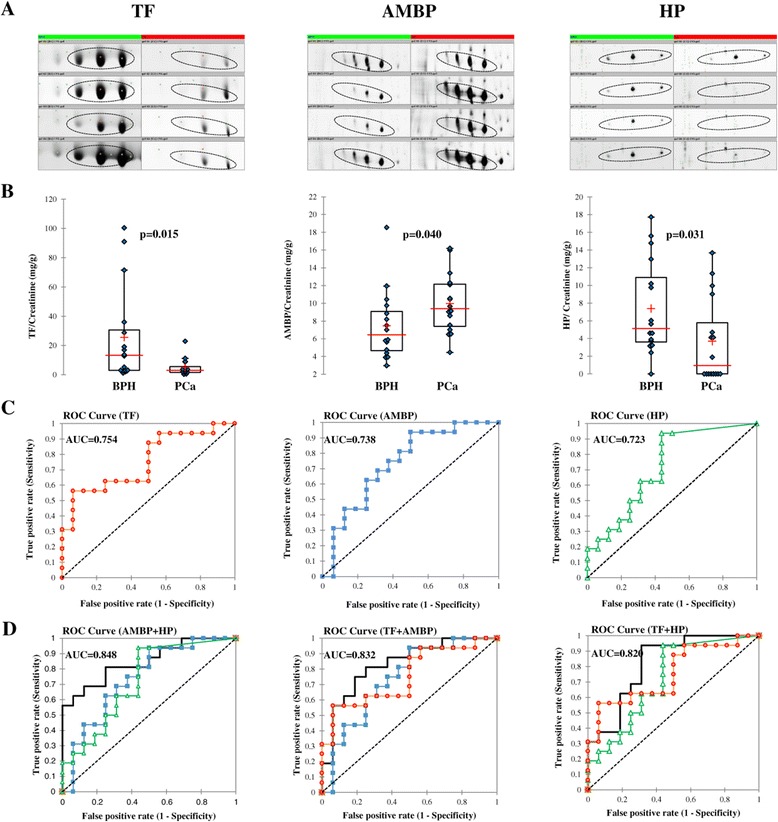
**Validation of candidate biomarkers for the diagnosis of PCa. (A)** 2-DE profiles of TF, AMBP and HP abundance in independent urine samples from BPH and PCa patients obtained by 2-D DIGE. Proteins with differential abundance were represented by clusters of 3–4 spots, highlighted with oval lines. Four gels corresponding to samples from each group were shown. **(B)** TF, AMBP and HP levels in urine of PCa and BPH patients, expressed as relative ratio to urine creatinine and obtained by immunoturbidimetry. AMBP level in PCa was significantly higher than that in BPH while TF and HP levels in PCa were significantly lower than in BPH (Mann–Whitney *U*-test, P < 0.05). In the combined dot/box plot graphs, concentration data (blue diamond), median (−), 25th and 75th percentiles and mean (+) are shown. **(C)** Urinary TF, AMBP and HP distinguish PCa on independent series of urine samples from patients with PCa and BPH. The optimal cutoffs for the proteins were: 12.81 mg TF/g creatinine (93.8% specificity, 56.3% sensitivity); 6.51 mg AMBP/g creatinine (50.0% specificity, 93.8% sensitivity); 2.40 mg HP/g creatinine (56.3% specificity, 93.8% sensitivity). ROC curves were based on series of 32 urine samples. **(D)** The diagnostic accuracy of TF, AMBP and HP combinations using logistic regression model. The combination AMBP and HP yielded highest diagnostic accuracy (AUC = 0.848).

Five proteins (TF, SERPINA1, APOA1, AMBP and HP) were further evaluated by immunoturbidimetry to test whether quantitative measurement in urine could be utilized as a diagnostic tool to distinguish patients with PCa and BPH. We manage to determine quantitatively the urine levels of three proteins (TF, AMPB and HP), while the concentrations of SERPINA1 and APOA1 were below the assays LOD. The measured concentrations of TF, AMPB and HP were normalized to urine creatinine to make correction for variations in urinary concentration. The results confirmed the abundance levels obtained by the DIGE experiment (Figure 4A). The concentration of AMPB in the PCa group showed a significantly higher level that in BPH group (Mann–Whitney *U*-test, p = 0.04). The levels of TF and HP were significantly higher in the BPH compared to the PCa group yielding p = 0.015 and p = 0.031, respectively (Figure 4B). Receiver operating curve (ROC) determined the diagnostic accuracy of the proteins in the validation set, using the histopathological results as the gold standard for clinical diagnosis and classification (Figure 4C). The area under the curve (AUC) for TF was 0.754 (p = 0.002, 95% CI 0.596-0.912), for AMBP was 0.738 (p = 0.005, 95% CI 0.574-0.903) and for HP was 0.723 (p = 0.008, 95% CI 0.558-0.888). The optimal cutoffs for the proteins were: 12.81 mg TF/g creatinine (93.8% specificity, 56.3% sensitivity); 6.51 mg AMBP/g creatinine (50.0% specificity, 93.8% sensitivity); 2.40 mg HP/g creatinine (56.3% specificity, 93.8% sensitivity). The values of AUC and diagnostics cutoff for serum PSA in the validation set were 0.754 (p = 0.001, 95% CI 0.600-0.907) and 5.50 ng/ml (50.0% specificity, 93.8% sensitivity), respectively (data not shown).

Using a logistic regression model, the diagnostic accuracy of various combinations of TF, AMBP, HP and PSA were tested (Table 3 and Additional file 2). The combination of TF, AMBP and HP increased the individual diagnostic accuracy (Figure 4D). The highest AUC was obtained for the combination of HP and AMBP (AUC = 0.848). Inclusion of PSA or TF into the HP/AMBP test has not yielded an improved accuracy. The combination of each of the tested proteins with PSA also resulted in increased AUC, except for the AMBP where addition of PSA did not improve the diagnostic accuracy (0.773 (AMBP + PSA) vs. 0.738 (AMBP) vs 0.754 (PSA).

**Table 3:** **The diagnostic accuracy of various combinations of TF, AMBP, HP and PSA expressed as area under the ROC curve (AUC) using logistic regression model**

	**Protein combinations tested**								
TF	**√**	**√**			**√**	**√**	**√**		
HP	**√**	**√**	**√**	**√**		**√**		**√**	
AMBP	**√**	**√**	**√**	**√**	**√**				**√**
PSA	**√**		**√**				**√**	**√**	**√**
AUC	0.840	0.848	0.848	0.848	0.832	0.820	0.840	0.836	0.773

## Discussion

The low sensitivity and specificity of current diagnostic methods for prostate cancer highlights the need for improvement in this area. In this study, we focused on identification of non-invasive biomarkers for PCa using urine samples from two age-matched groups of patients with histologically characterized diagnosis of PCa and BPH, respectively.

Urine is an attractive material in clinical proteomics because it can be sampled non-invasively in large quantities, contains generally soluble proteins which does not undergo significant proteolytic degradation and has lower dynamics range of protein concentrations compared to other biofluids [40]. Urine proteome represents modified ultrafiltrate of plasma combined with proteins derived from kidney and urinary tract [41]. Proteomic analysis of urine has suggested that it contains disease-specific information for a number of kidney diseases, cancers related to the urogenital system such as kidney, bladder and prostate cancer, as well as various non-nephrological/urogenital diseases [42]. The comparative proteomics studies for PCa biomarker identification in urine have reported a number of proteins with differential abundance between PCa and BPH, with some of them proposed as potential biomarkers for PCa diagnosis [22-27,43,44]. However, most of these candidate biomarkers still lack an extensive validation and haven’t been introduced into clinical practice. The high variability of the reported data so far highlights the need for more research in this area with consistent sample collection, storage methods and sample processing. The samples used in this study were collected and stored according to the standard protocol for urine collection with no addition of protease and phosphatase inhibitors and no pH adjustment [45]. Sample processing and manipulation were minimal without depletion of the highly abundant proteins, to exclude the possibility of losing low abundant proteins or low molecular weight proteins that exist in complexes with the highly abundant proteins. In addition, we have used the 2-D DIGE/MS platform who although has lover dynamic range of protein quantification than LC-MS based platforms is still an indispensable platform in proteomics, particularly for the visualization of the proteome and assessment of the individual posttranslational modifications [46].

Our proteomic data revealed 23 proteins with differential abundance in urine of PCa patients compared to BPH patients. In order to analyze the impact of co-regulation of protein expression in context of biomarker identification, principal component analysis and hierarchical clustering has been applied on the differential protein expression data. Here, we have detected a clear separation of the two distinct groups (PCa and BPH) based on the abundance pattern of the 23 proteins. The clustering of samples showed formation of two separate clusters, clearly separating benign from tumor samples with general observation that PCa/BPH classification cannot be based on one individual protein, but on expression pattern of the selected subset of proteins.

Gene Ontology (GO) search for biological processes classified these proteins into proteins involved in the immune response and response to stimuli, regulators of different biological processes (transcription, proliferation, signal transduction, cytokine production), transport proteins involved in transport of ions, proteins and lipids and proteins involved into different metabolic processes.

Many of the proteins with differential abundance in this study have been associated with PCa or cancers of the urogenital system (Table 2). Overall, 17 of the identified proteins have been associated specifically with PCa in different proteomics studies. Our initial study of the protein components from urine of PCa patients, pointed out 11 proteins that might have some role in the pathogenesis of prostate cancer by comparison with other published studies analyzing normal urine proteome [28]. Five of these proteins (TYMP, ENDOD1, RFFL, CRYZL1 and ILF2) were detected with differential abundance when comparing to BPH group. In comparative studies using urine, the abundance levels of TF, ITIH4, AMPB, PTGDS, HP and UMOD were the same as in our study [23,26,29,38]. However, for ITIH4 and AMBP, there are also studies where opposite abundance levels were reported as well [31,38]. The abundance level of APOA1 in our study was the same as in a study using serum while the levels of SERPINA1 and TTR were found opposite to ours [31]. Some of the proteins such as SERPINA1, FGG, FGA, APOA1 and HP were also found with differential abundance in urine in proteomics studies of bladder cancer, and TYMP in tissue in renal cell carcinoma, but mostly with opposite abundance level compared to our study [32,34-36].

IPA analysis of our set of proteins revealed significant association with the Acute Phase Response Signaling pathway. Nine of our proteins (AMBP, APOA1, FGA, FGG, HP, ITIH4, SERPINA1, TF and TTR) were acute phase response proteins. The acute phase response is a rapid inflammatory response that provides protection against microorganisms using non-specific defense mechanisms [47]. The association of our proteins with this pathway complies with the generally accepted observation that inflammation is often observed in tumors and appears to play a dominant role in the pathogenesis of various cancer types [48]. Moreover, the highest ranked protein network of functional associations between the differentially expressed proteins revealed close connected in the network through three major nodes: P38 Mitogen activated protein kinase (P38 MARK), extracellular-signal-regulated kinase 1/2 (ERK1/2) and pro-inflamatory cytokines. P38 MARK and ERK1/2 are members of the mitogen activated protein kinase super family that can mediate cell proliferation and apoptosis [49]. Abnormal regulation of the MAPK pathways have been reported for a wide range of diseases including many cancers [50]. The pro-inflamatory cytokines interleukin-6 (IL-6), tumor necrosis factor alpha (TNFα) and interleukin-1 beta (IL-1β) are critical mediators of the systemic inflammatory response and main stimulators for the synthesis of an acute-phase response proteins [48]. These cytokines have been implicated in a variety of diseases including cancer where their role is more likely to contribute to tumour growth, progression and immunosuppression than to an effective host-antitumor response [51]. For this reason, cancer patients frequently present changes in various systemic parameters, comprising alterations in the level of serum inflammatory cytokines, acute-phase proteins and total albumin [52]. And finally, IPA biomarker filter revealed 12 proteins as candidate biomarkers for prostate cancer with majority being acute phase proteins.

A number of well characterized acute phase proteins have been linked to distinct cancer types and stages of malignancy [53]. The field of acute phase proteins as cancer biomarkers has vast potential. The identification of specific proteomic expression patterns in acute phase proteins related to cancer offers promise for novel diagnostic markers. Having in mind this and results from the IPA analysis, we considered the possibility of using acute phase response proteins found in urine as non-invasive biomarkers for PCa. This was additionally encouraged from the fact that the abundance pattern of 4 acute phase response proteins in our study differed from the defined expression (Figure 4A). Protein AMBP shows decreased plasma concentration during the acute phase response, but in our study increased expression in PCa was observed. Proteins HP, SERPINA1 and FGG showed decreased expression in PCa, opposite to the plasma concentration during the acute phase response. On the other hand, the expression levels of the acute phase response proteins in this study correlated with the observed levels in a number of independent studies on PCa, with minor exceptions where opposite abundance levels were also reported (reviewed in Table 2).

From the total set of acute phase response proteins detected with differential abundance, we highlighted TF, AMBP, HP, APOA1 and SERPINA1. These proteins were detected as clusters of 2-6 spots with the same abundance levels within clusters and therefore exhibited higher potential to be associated with PCa than proteins identified in only one spot. Notably, AMBP, HP and SERPINA1 were particularly interesting for us since they demonstrated the same abundance levels as in other proteomics studies and opposite abundances from the acute phase response. So, we chose to further evaluate the diagnostic potential of these 5 proteins in an independent set of patients by quantitative measurement in urine. Using immunoturbidimetry, we manage to determine quantitatively the urine levels of TF, AMBP and HP in all samples from the validation set. The results showed that there was a significant difference (p<0.05) in the urine levels of these proteins between PCa and BPH group, confirming the expression levels obtained by the DIGE experiment. The observed diagnostics accuracy of the proteins was moderate, with the lowest being found for HP (0.723 at 2.40 mg/g creatinine) and the highest for TF (0.754 at 12.81 mg/g creatinine). Similar diagnostics accuracy was found for serum PSA (0.754). A number of logistic regression models using combinations of TF, AMPB, HP and PSA were also tested in order to estimate if the diagnostic accuracy improves with the combination of proteins. In general, combination of proteins showed increased AUC with the highest value obtained for HP/AMBP. So, although the observed accuracy of the individual proteins were similar to the PSA, the combination of HP and AMBP yielded greater accuracy compared to individual tests. The results indicated that HP/AMBP combination could be potential biomarker set for the diagnosis of PCa with improved accuracy compared to the PSA. In addition, the proposed biomarker set complies with the requirements for diagnostics biomarker as it is easily accessible, non-invasive, quickly quantifiable and economical. Though maybe these proteins are not specific proteins for PCa, they could become an important check index and improve the sensitivity and specificity for early diagnosis.

## Conclusions

Our study indicated that the 2-D DIGE/MS proteomic analysis of urinary proteins is feasible for the identification of non-invasive biomarkers for PCa diagnosis. As a result of this approach, a set of acute phase response proteins found in urine could serve as a diagnostics biomarkers for PCa. Moreover, our results confirmed the importance of previously identified proteins and highlighted new proteins that can add information regarding the pathophysiological mechanisms of PCa. However, further studies are needed to validate the proposed biomarkers in independent cohorts and to evaluate the diagnostic potential of the rest of the differentially expressed proteins found in this study which might further improve the diagnostics accuracy of the proposed set.

## Methods

### Samples

We analyzed 56 urine samples from patients with clinically and histological confirmed PCa and BPH obtained from the University Clinic for Urology, University Clinical Centre “Mother Theresa”, Skopje, Republic of Macedonia. Informed consent for the use of these samples for research purposes was obtained from the patients in accordance with the Declaration of Helsinki. The study has been approved by the Ethics Committee of the Macedonian Academy of Sciences and Arts.

The patients referred to hospital because of clinical symptoms. The diagnosis was based on histological evaluation of tissues obtained by transurethral resection of the prostate (TURP) for BPH and whole prostate gland obtained by radical prostatectomy for PCa patients. None of the patients received preoperative therapy. Patient’s clinical records including histology grading, tumor stage and pre-operative PSA were reviewed to preselect the urine samples used in this study (Additional file 3: Table S1). BPH patients were preselected to be without signs of inflammation (prostatitis). Urine samples for the 2D-DIGE analysis (screening set) consisted of 8 samples from PCa and 16 samples from BPH patients. Validation of the selected candidate biomarkers was done on an additional 16 PCa and 16 BPH urine samples. In the screening cohort, the mean values (± SD) for PSA, Gleason score and age were as follows: serum PSA was 9.0 ± 3.9 (ng/ml) for the PCa group and 6.4 ± 3.2 (ng/ml) for the BPH group; age was 69.0 ± 6.6 years for PCa and 65.3 ± 7.3 years for BPH; Gleason score was 7.4 ± 1.1. In the validation cohort, the mean values (± SD) for PSA, Gleason score and age were as follows: serum PSA was 8.7 ± 3.2 (ng/ml) for PCa group and 5.6 ± 2.9 (ng/ml) for BPH group; age was 67.4 ± 5.0 years for PCa and 69.2 ± 8.2 years for BPH; Gleason score was 7.4 ± 1.1.

The first morning urine (3–10) ml was collected from the patients prior to clinical intervention and stored on ice for short period (<1 h). Samples were centrifuged at 1000 g, for 10 min to remove cell debris and casts, aliquoted in 1.5 ml tubes and stored at −80°C until use.

The stored urine samples were thawed and for each sample, proteins were isolated in triplicate from 100 μl urine using 2-D Clean-UP Kit (GE Healthcare) according to the manufacturer’s instructions. The pellets from each replicate were dissolved in 10 μl of UTC buffer (8 M Urea, 2 M Thiourea, 4% CHAPS), pooled together for each sample, quantified by the Bradford method [54] in duplicate against a standard curve of Bovine Serum Albumin (BSA) and stored at −80°C until use.

### 2-D DIGE, imaging and analysis

Equal amounts of protein extract from urine were pooled for the DIGE labeling: 2 PCA samples and 4 BPH samples per labeling reaction respectively. The pH of protein samples was adjusted to 8.5 with 1.5 M Tris–HCl. Proteins were labeled with the CyDye DIGE Fluor minimal dyes (GE Healthcare) following manufacturer’s instructions. Forty five micrograms of protein per pool were minimally labeled with 400 pmol of Cy3 or Cy5, respectively. Cy2 was used to label an equivalent amount of internal standard containing equal amounts of all samples. Reactions were stopped with 10 mM L-lysine for 10 min. The samples were randomized between gels to ensure an even distribution between those labelled with Cy3 and Cy5 minimal dyes and to avoid repetitive linking of the same sample type with the same dye on multiple gels.

The first dimension of the 2-D DIGE analysis was performed using 24 cm Immobiline Drystrip gels (GE Healthcare) with linear pH 4–7 gradient. The separate CyDyes labeling reactions were combined, rehydration buffer (8 M Urea, 2 M Thiourea, 2% (w/v) CHAPS, 10 mM DTT, 1.2% (v/v) IPG-Buffer pH4-7, Trace of Bromophenol Blue) was added to a final volume of 450 μl and the gels were passively rehydratated overnight in IPGPhor cassettes. Isoelectric focusing (IEF) was performed on the Ettan IPGphor 3 system (GE Helthcare) under the following conditions: 3 h at 300 V, 7 h gradient to 1000 V, 3 h gradient to 10000 V and 4 h 15 min at 10000 V, until total a of 64.5 kVh was reached. The focused proteins in the IPG strips were immediately equilibrated in two incubation steps, each lasting 15 min, at room temperature. In the first step, the equilibration buffer (6 M Urea, 2% SDS, 30% Glycerol, 50 mM Tris–HCl, pH 8.6) was supplemented with 1% (w/v) DTT for reduction, followed by alkylation in the same buffer containing 4.7% (w/v) iodoacetamide instead of DTT. The second dimension was carried out onto 12.5% homogeneous polyacrylamide gels using the Ettan DALTsix system (GE Healthcare), at 2.5 W per gel for 30 min, followed by 16 W/gel for 5 h.

The four 2-D DIGE gels were scanned on an Ettan DIGE imager (GE Healthcare). Gel images were normalized by adjusting the exposure time to obtain appropriate pixel value without any saturation. All gels were scanned at 100 dpi resolution. Images were cropped using Image QuantTL software v7.0 (GE Healthcare) to remove areas extraneous to the gel image.

DIGE images were analyzed using DeCyder 2-D Differential Analysis Software v7.1 (GE Healthcare). Spot detection and normalization was processed by the Differential Analysis (DIA) module using the estimated number of spots set to 3000 and spot volume < 30000 as exclusion filter. Gel-to-gel comparison and statistical analysis of the degree of difference in standardized protein abundance between PCa and BPH groups were performed with the Biological Variation Analysis (BVA) module. Matching was further improved by using landmarks and manually confirming potential spots of interest. Proteins with statistically significant differential abundance were selected based on two criteria: *t*-test < 0.05 and ratio > 1.8. Each spot was manually verified for an acceptable three dimensional characteristic protein profile and for adequate material for subsequent mass spectrometry identification. Spots not meeting these criteria were excluded from further analysis. The Extended Data Analysis (EDA) module of DeCyder and was used for principal component analysis and clustering studies. Selected protein spots with significant difference were excised from a preparative gel stained with CBB G-250 and identified by MALDI MS.

### Setting up of preparative 2-D gels for spot picking

For preparative Coomassie brilliant blue (CBB) - stained gel, 60 μg of each of the 8 protein pools were combined, to give a total of 480 μg of protein. The volume was adjusted to 450 μl with the rehydration buffer. The IEF and second dimension SDS-PAGE were run according to standard procedures. Gel was fixed in 30% (v/v) ethanol, 2% (v/v) phosphoric acid for 30 min with two exchanges of the fixing solution, washed three times with 2% (v/v) phosphoric acid for 10 min each, balanced in pre-staining buffer (12% (w/v) (NH_4_)_2_SO_4,_ 2% (v/v) phosphoric acid, 18% (v/v) ethanol) for another 30 min and stained in staining solution (0.01% (w/v) CBB G-250, 12% (w/v) (NH_4_)_2_SO_4,_ 2% (v/v) phosphoric acid, 18% (v/v) ethanol) for 72 h. The gel was stored in the staining solution until the spots of interests were manually picked.

### Mass spectrometry: in-gel tryptic digestion and identification

In-gel digestion was carried out manually with trypsin. Spots were first destained twice with a mixture of 50% (v/v) ACN for 15 min each and than once with 100 mM NH_4_HCO_3_ and 50% (v/v) ACN for 15 min. Spots were dried in vacuum centrifuge and then reduced with 100 mM NH_4_HCO_3_ containing 10 mM DTT for 45 min at 56°C, and then alkylated with 54 mM iodoacetamide in 100 mM NH_4_HCO_3_ for 30 min in the dark, at room temperature. Gels pieces were washed with 100 mM NH_4_HCO_3_, shrunk with 50% ACN for 15 min and dried in a vacuum centrifuge. Gel particles were rehydrated with 20 μl of 0.01 μg/μl trypsin proteomics grade (Roche Diagnostics GmbH) in digestion buffer (95% 50 mM NH_4_HCO_3_/5% ACN) for 45 min at room temperature. The remaining enzyme supernatant was replaced with one gel volume of the digestion buffer and digestion was carried out at 37°C, overnight. After digestion, peptides were collected in a separate tube, extracted once with 20 μl of 50% ACN and twice with a mixture of 50% ACN/5% formic acid, dried in a vacuum centrifuge and reconstituted in 10 μl of 0.1% TFA.

For MS analysis, peptides were purified using ZipTip_C18_ (Millipore Corporation) following the manufacturer’s instructions and eluted in 2–3 μl of CHCA (4 mg/ml in 50% ACN/0.1% TFA) directly onto a MALDI target plate (Shimadzu Biotech Kratos Analytical). Droplets were allowed to dry at room temperature. Samples analysis was performed using AXIMA Performance MALDI-TOF-TOF mass spectrometer (Shimadzu Biotech Kratos Analytical). Spectra acquisition and processing was performed using the MALDI-MS software (Shimadzu Biotech Kratos Analytical) version 2.9.3.20110624 in positive reflectron mode at mass range 1–5000 Da with a low mass gate at 500 Da and pulsed extraction optimized at 2300 Da. External calibration was performed based on monoisotopic values of five well-defined peptides: Bradykinin fragment 1–5, Angiotensin II human, Glu1-Fibrinopeptide B human, Adrenocorticotropic Hormone Fragment 1–17 human and Adrenocorticotropic Hormone Fragment 7–38 human (Sigma-Aldrich). External calibration mix (500 fmol/μl) was diluted with the matrix in ratio 1:1 and applied onto the MALDI target plate at final concentration of 250 fmol per spot. Each mass spectrum was acquired by 500 laser profiles (five pulses per profile) collected across the whole sample.

After filtering tryptic-, keratin- and matrix-contaminant peaks, the resulting monoisotopic list of m/z values was submitted to the search engine MASCOT (version 2.4.01, MatrixScience, UK) searching all human proteins and sequence information from Swiss-Prot (version 2014_05, 20265 sequences) and NCBInr (version 20140323, 276505 sequences). The following search parameters were applied: fixed modification-carbamidomethylation, variable modifications-methionine oxidation and N-terminal acetylation. Up to 1 missed tryptic cleavage was permitted and a peptide mass tolerance of ±0.40 Da was used for all mass searches. Positive identification was based on a Mascot score greater than 56, above the significance level (p < 0.05). The reported proteins were always those with the highest number of peptide matches.

### Quantitative determination of selected proteins in urine

The concentrations of Serotransferrin, α1- antitrypsin, α1- microglobulin/bikunin, Apolipoprotein A-I and Haptoglobin in urine were measured by immunoturbidimetry using COBAS Integra 400 Plus (Roche Diagnostics). The kits used for measurement of these proteins in urine and detection limits were as follows: Serotransferrin, TQ Transferrin ver. 2 (Roche Diagnostics) with LOD = 0.013 g/L; α1- antitrypsin, TQ a-1 Antitrypsin ver. 2 (Roche Diagnostics) with LOD = 0.2 g/L; α1-microglobulin/ bikunin, TQ a-1 Microglobulin Gen. 2 (Roche Diagnostics) with LOD = 5 mg/L; Apolipoprotein A-I, TQ APO A-1 ver. 2 (Roche Diagnostics) with LOD = 0.2 g/L; Haptoglobin, TQ Haptoglobin ver. 2 (Roche Diagnostics) with LOD = 0.1 g/L. The level of creatinine in urine was determined using the Jaffé method by Crea Jaffe Gen. 2 Urine Kit (Roche Diagnostics) with 0.01 mmol/l limit of detection.

### Bioinformatics and statistical analysis of the proteomics data

For an overview of the cellular localization, molecular function and biological processes in which identified proteins are included in, we used the UniProt Knowledgebase (UniProtKB) and Gene Ontology (GO) database. Pathway analysis was carried out for proteins found to be differently expressed between tumor and control samples using Ingenuity Pathway Analysis (IPA) (Ingenuity Systems, USA). Identified proteins were functionally assigned to canonical pathways and sub sequentially mapped to the most significant networks generated from previous publications and public protein interaction databases. A *p* value calculated with the right-tailed Fisher’s exact test was used to yield a network’s score and to rank networks according to their degree of association with our data set.

The Mann–Whitney *U*-test was used to analyze the correlation between the levels of the 3 selected proteins in urine and the pathological and clinical stage of prostate. The relative effectiveness of the diagnostic tests was illustrated by plotting the true-positive (sensitivity) versus the false-positive (1-specificity) results in receiver operating characteristic (ROC) curves. ROC curve analyses were used to define the most optimal diagnostic cutoff as well as the diagnostic performance given by areas under the curves (AUC). AUC were compared using a nonparametric method as described by Bamber [55]. In order to estimate the combined diagnostic potential of several candidate biomarkers, logistic regression analyses were performed with clinical diagnosis (PCa/BPH) as dependent variable and measurement of the protein concentrations in urine of selected proteins as independent variables. A confidence level of 95% (p < 0.05) was considered significant for all performed tests. Statistical analyses were performed using XLSTAT software (ver. 2014.4.06).

## Additional files

Additional file 1: Figure S1. Classification of urine proteins with differential abundance between PCa and BPH. The molecular function, biological processes in which they are involved, subcellular location and type of the proteins were assessed by Gene Ontology search. The numbers represent percentages. Additional file 2:
**Logistic regression models and ROC curves of combinations of TF, AMPB, HP and PSA. **The summary of these results is presented in Table 3. Additional file 3: Table S1. Clinical information of patients used to generate urine samples included in the study together with their PSA levels, histology grading and tumor stage.

## Abbreviations

2-D: Two-dimensional
2-D DIGE: Two-dimensional difference in gel polyacrylamide gel electrophoresis
ACN: Acetonitrile
AUC: Areas under the curves
BPH: Benign prostatic hyperplasia
BSA: Bovine serum albumin
BVA: Biological variation analysis
CHAPS: 3-[(3-Cholamidopropyl) dimethylammonio-1-propanesulfonate
CBB: Coomassie brilliant blue
Da: Dalton
DIA: Differential analysis
DTT: Dithiothreitol
GO: Gene ontology
IAA: 2-Iodoacetamide
IPA: Ingenuity pathways analysis
MALDI-TOF-TOF: Matrix-assisted laser desorption/ionization-time of flight-time of flight
MS: Mass spectrometry
PCa: Prostate cancer
PSA: Prostate-specific antigen
ROC: receiver operating characteristic
SDS: Sodium dodecyl sulfate
SDS-PAGE: Sodium dodecyl sulfate polyacrylamide gel electrophoresis
TFA: Trifluoroacetic acid
Tris: Tris(hydroxymethyl)amino
