# Supplementary figures and images for: Proteomics analysis of urine reveals acute phase response proteins as candidate diagnostic biomarkers for prostate cancer
Source: Proteome Sci. 2015 Jan 29;13:2. doi: 10.1186/s12953-014-0059-9 (PMC4316650; doi:10.1186/s12953-014-0059-9)

## Slide 1
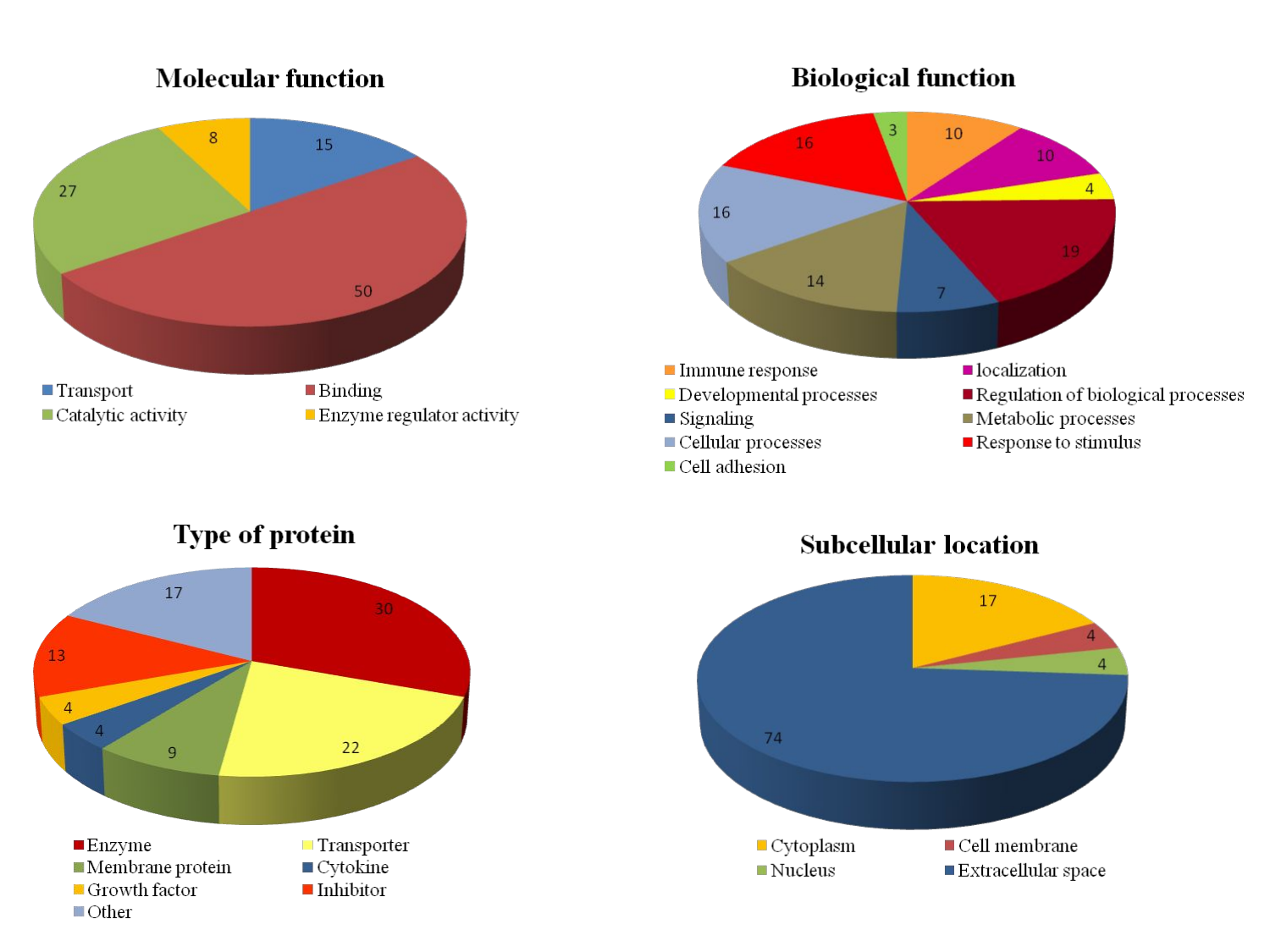

Supplement: Additional file 1: Figure S1. — Classification of urine proteins with differential abundance between PCa and BPH. The molecular function, biological processes in which they are involved, subcellular location and type of the proteins were assessed by Gene Ontology search. The numbers represent percentages. [file 12953_2014_59_MOESM1_ESM.ppt]
